# Analysis of factors that increase serum progesterone levels on the day of hCG administration in gonadotropin-releasing hormone antagonist cycles

**DOI:** 10.3389/fendo.2026.1766613

**Published:** 2026-06-09

**Authors:** Jingwen Wang, Xu Liu, Jiankang Xue, Youzhong Zhang

**Affiliations:** 1 Department of Obstetrics and Gynecology, QiLu Hospital of Shandong University, Jinan, Shandong, China; 2 Center for Reproductive Medicine, Department of Obstetrics and Gynecology, Affiliated Hospital of Jining Medical University, Jining, Shandong, China; 3 Department of Gynecology II, Tengzhou Central People’s Hospital, Zaozhuang, Shandong, China; 4 Jining Medical University Clinical Medical College, Jining, China

**Keywords:** controlled ovulation stimulation, gonadotropin-releasing hormone antagonist, HCG day, *in vitro* fertilization, progesterone

## Abstract

**Objective:**

To explore the factors associated with elevated serum progesterone (P) levels on the day of hCG administration in patients undergoing controlled ovarian stimulation using a gonadotropin-releasing hormone antagonist (GnRH-A) protocol.

**Methods:**

A retrospective cohort study was conducted, including 1392 *in vitro* fertilization-embryo transfer (IVF-ET) or intracytoplasmic sperm injection (ICSI) cycles performed between January 2018 and December 2023 at the Reproductive Medicine Center of the Affiliated Hospital of Jining Medical University. Patients were stratified into a normal progesterone group (NP group, serum P ≤ 1.5 ng/mL, n = 985 cycles) and a high progesterone group (HP group, serum P > 1.5 ng/mL, n = 407 cycles) according to the cutoff value of 1.5 ng/mL on hCG day. Clinical characteristics and endocrine parameters during ovarian stimulation were compared between groups. Univariate and multivariate logistic regression analyses were applied to identify independent factors related to elevated hCG-day progesterone levels.

**Results:**

The oocyte retrieval age, BMI, basal FSH (bFSH), and hCG-day serum LH levels were significantly higher in the NP group than in the HP group, whereas basal P (bP), antral follicle count, hCG-day serum E2, AMH, and total number of oocytes retrieved were significantly lower (all P < 0.05). No significant differences were observed in basal LH, basal E2, initial Gn dose, total Gn duration, or total Gn dose between groups (all P > 0.05). Univariate logistic regression showed that oocyte retrieval age, BMI, bFSH, bP, AMH, antral follicle count, hCG-day serum LH and E2 levels, and total oocytes retrieved were significantly associated with hCG-day progesterone levels (all P < 0.05). Multivariate analysis further confirmed that BMI (OR = 0.901), bP (OR = 2.100), hCG-day serum E2 (OR = 1.225), hCG-day serum LH (OR = 1.048), and total oocytes retrieved (OR = 1.143) were independent influencing factors (all P < 0.05).

**Conclusion:**

Basal progesterone, hCG-day serum E2 and LH levels, and total number of oocytes retrieved are independent risk factors for elevated progesterone levels on hCG day, whereas BMI serves as a protective factor.

## 1 Introduction

Currently, assisted reproductive technology (ART) has become the most effective method to treat infertility. Controlled ovulation stimulation is a critical step of assisted reproductive technology. Compared with frozen embryo transfer (FET), fresh embryo transfer has lower adverse pregnancy outcomes, such as gestational hypertension and preeclampsia, and FET is related to the degree of gestational hypertension (1, 2). The relevant mechanism is still unclear and may be related to abnormal placental anatomy or vascular lesions (3). In 1999, the gonadotropin-releasing hormone agonist (GnRH-A) was introduced. As time goes by, studies have shown that the GnRH-A regimen can effectively suppress premature ovulation compared with the GnRH-A regimen. The LH peak is generated to prevent premature discharge of follicles, and the risk of ovarian hyperstimulation syndrome (OHSS) is lower (4). The endometrium is closer to its natural state (5). The GnRH-A program is safer and more cost-effective (6), and it has become one of the mainstream options for ovulation induction.

The probability of an increase in serum progesterone (P) level on the HCG day is 12% to 38%. On the one hand, it affects the endometrium and the implantation window. The “implantation window” opens in advance, causing the endometrium and embryonic development to be out of sync (7). On the other hand, it affects oocytes quality, which in turn affects embryos quality, ultimately leading to a decrease in the pregnancy rate (8). At present, a large number of studies have founded that there was a “threshold effect” (0.8–2.0 ng/mL) in the serum P level on HCG day. If it exceeds the “threshold”, the pregnancy rate/clinical pregnancy rate/cumulative pregnancy rate will show a downward trend (9). Unfortunately, the mechanism responsible for the increase in serum P levels on hCG day is not yet clear, and may be related to the process of controlled ovarian stimulation (10). The purpose of this study is to explore the factors that cause an increase in serum progesterone (P) levels on HCG days during controlled ovarian stimulation in infertile individuals using the GnRH-A regimen, and to assist clinicians in adjusting strategies in a timely manner to reduce serum P levels on HCG days and improve clinical outcomes.

## 2 Materials and methods

A total of 1392 cycles were included in this study. Consistent with previous large-scale studies and meta-analyses (7, 11, 12), the cutoff value for serum progesterone on the day of hCG administration was set at 1.5 ng/mL, as this threshold has been widely reported to be associated with reproductive outcomes in GnRH antagonist cycles. And divided into 2 groups, the normal group (NP) (P ≤ 1.5ng/ml), with a total of 985 cycles, and the high-level group (HP) (P>1.5ng/ml), with a total of 407 cycles. Single-factor logistic regression analysis was performed on the indicators related to the increase in serum P level on HCG day. The results showed that Maternal age, BMI, bFSH, bP, number of antral follicles, serum LH and E2 levels on the HCG day, and the total number of oocytes retrieved were all related to the serum P level on the HCG day (*p* < 0.05). Multivariate logistic regression analysis was further performed to determine the predictors of increased serum P levels on HCG days.

### 2.1 Study design

This retrospective study examined patients who underwent *in vitro* fertilization-embryo transfer (IVF-ET) or intracytoplasmic sperm injection (ICSI) from January 2018 to December 2023 at the Department of Reproductive Medicine, Affiliated Hospital of Jining Medical University. Infertile patients aged 20–40 years old who used antagonist regimens to induce ovulation and underwent IVF/ICSI/IVF+ICSI fresh embryo transfer to assist pregnancy in our center were included. Exclusions include diagnosis of polycystic ovary syndrome, presence of ovarian cysts on one or both sides, refusal of fresh embryo transfer, use of other programs to induce ovulation, failure to retrieve oocytes on the day of oocyte retrieval, and patients who discontinue the process due to other diseases during treatment. The study protocol was approved by the Institutional Review Board of the hospital (The ethical review number: 2025-09-C004).

### 2.2 Ovarian stimulation protocols

According to the patient’s age, body mass index (BMI), ovarian reserve, and other factors, the type and dose of gonadotropin (Gn) can be flexibly selected to induce ovulation on the 2nd to 3rd day of menstruation. Gn includes Gonal-F (33μg, Merck Serono, Germany) or urinary gonadotropin (75U, China Livzon Pharmaceutical Group Co., Ltd.). During the ovulation induction process, regular vaginal ultrasound is used to monitor follicular development and adjust drug dosage. When the diameter of the dominant follicle reaches 12 to 14 mm, Cetrotide (specification: 0.25 mg, Merck Serono, Germany) is added.

### 2.3 Trigger and oocyte retrieval

When vaginal ultrasound monitors that three follicles are 18 to 20 mm in diameter, the patient will be given intramuscular injection of human chorionic gonadotropin (hCG) (specification: 5000 to 10000IU, China Livzon Pharmaceutical Group) Co., Ltd.); or recombinant human chorionic gonadotropin, rhCG (specification: 250 μg, Merck Serono, Germany). At the same time, a gonadotropin-releasing hormone agonist, GnRH-a (specification: 0.1 mg triptorelin, Ferring GmbH, Germany), was given. For the trigger or double trigger, an injection of 2000–4000 IU of hCG was injected subcutaneously. And follicles were collected under vaginal ultrasound guidance 36 to 38 hours later. Finally, count the number of punctured follicles and count the number of harvested follicles.

### 2.4 Observation indicators

On days 2 to 4 of the menstrual period, a fully automatic chemiluminescence immunoassay analyzer (Beckman DIX-800) was used to measure basic sex hormone levels and HCG day-related hormone levels. The observation indicators are the patient’s baseline information, including age, BMI, years of infertility, type of infertility, infertility factors, reproductive endocrine levels (bFSH, bLH, bE2, bP, and AMH), number of antral follicles, ovulation induction status, including Gn Initial dose, number of Gn days, total Gn dose, endometrial thickness on HCG day, E2 level on HCG day, LH level on HCG day, and total number of retrieved eggs.

### 2.5 Statistical analysis

This study used SPSS 26.0 statistical software for data analysis. Measurement data were statistically described using mean or median (interquartile range) [M (P25, P75)], and the t-test or the non-parametric test was used for comparison between groups. Count data were expressed as the percentage (%), and the chi-square test was used for comparison between groups. Single-factor logistic regression analysis was performed on related indicators that may affect clinical outcomes to further determine the confounding factors that affect the increase in progesterone levels on HCG days. Then, a multi-factor logistic regression analysis was performed to obtain meaningful factors that affect the increase in progesterone levels on HCG days. *p* < 0.05 means the difference is statistically significant.

## 3 Results

### 3.1 Patient demographic and cycle characteristics

In this study, the incidence rate of HCG day serum P level exceeding the threshold (1.5 ng/ml) was 29.24% (407/1392 cycles). As shown in **Table 1**, the HP group’s bLH, bE2, Gn initial dose, total Gn days, total Gn dose and AMH were all higher than those of the NP group. The number of years of infertility and endometrial thickness on HCG days were higher in the HP group than in the NP group, but the differences were not statistically significant (*p*>0.05). The bFSH, egg retrieval age, BMI, and serum LH levels on HCG day in the NP group were higher than those in the HP group. However, the bP, AMH, antral follicle number, serum E2 on HCG day and total number of retrieved eggs were lower in the NP group, and the differences were statistically significant (*p* < 0.05).

**Table 1:** Patient demographic and cycle characteristics.

	NP group	HP group	*P*
Maternal age (y)	35.39±5.41	33.46±4.93	<0.01^*^
BMI (kg/m2)	23.70±3.44	22.56±3.01	<0.01^*^
Infertility duration (y)	2.00(1,4)	2.50(1,4)	0.509
Type of infertility			0.003^*^
Primary infertility	255(25.9%)	137(33.7%)	
Secondary infertility	730(74.1%)	270(66.3%)	
Infertility diagnosis			0.897
PCOS	12(1.2%)	3(0.7%)	
DOR	22(2.2%)	6(1.6%)	
Tubal	367(37.3%)	147(36.1%)	
Endometriosis	6(0.6%)	1(0.2%)	
multifactor	33(3.4%)	13(3.2%)	
Male factors	202(20.5%)	88(21.6%)	
Mutual factors	211(21.4%)	94(23.1%)	
Unexplainable reasons	132(13.4%)	55(13.5%)	
Basal FSH (mIU/mL)	9.00±3.43	8.06±2.44	<0.01^*^
Basal LH (mIU/mL)	4.55±2.25	4.79±2.55	0.092
Basal E2 (pg/ml)	42.17±22.17	43.58±19.28	0.261
Basal P (ng/ml)	0.56±0.33	0.66±0.38	<0.01^*^
AMH(ng/ml)	2.13±1.94	3.48±2.67	<0.01^*^
No. Of antral follicles	10.27±6.55	14.68±6.82	<0.01^*^
Gn initial dose (IU)	248.26±63.06	250.43±64.32	0.565
Gn total dose (IU)	2755.31±946.23	2818.61±872.66	0.246
Gn duration (day)	10.71±2.06	10.91±1.74	0.058
hCG day LH (IU/L)	2.41(1.49,4.00)	1.85(0.96,2.88)	<0.01^*^
hCG day E2 (pg/ml)	2358.47(1221.16,3969.33)	4864.6(2659.05,6856.27)	<0.01^*^
hCG day P (ng/ml)	0.85±0.35	2.28±1.14	<0.01^*^
Endometrial thickness (mm)	10.45±2.95	10.22±2.54	0.159
No. Of retrieved oocytes	6.38±4.29	10.96±5.43	<0.01^*^

DOR, Diminished ovarian reserve.

Data are presented as mean ± standard deviation or number (%).

P < 0.05 is considered to be statistically significant.

### 3.2 Univariate analysis

Single-factor logistic regression analysis was performed on the indicators related to the increase in serum P level on HCG day. The results in **Table 2** show that the age of oocyte retrieval, BMI, bFSH, bP, AMH, number of antral follicles, serum LH and E2 levels on the HCG day and the total number of oocytes retrieved are all related to the serum P level on the HCG day (*p* < 0.05).

**Table 2:** Single-factor logistic regression analysis.

	β	SE	Waldχ^2^	*P*	OR(95%CI)
Maternal age (y)	-0.069	0.011	36.419	<0.01^*^	0.934(0.913-0.955)
BMI (kg/m2)	-0.111	0.019	32.811	<0.01^*^	0.895(0.862-0.93)
Infertility duration (y)	-0.027	0.024	1.256	0.262	0.973(0.929-1.02)
Type of infertility	0.373	0.128	8.554	0.003^*^	1.453(1.131-1.865)
Basal FSH (IU//L)	-0.114	0.023	25.188	<0.01^*^	0.892(0.853-0.933)
Basal LH (IU/L)	0.041	0.024	2.824	0.093	1.042(0.993-1.093)
Basal E2 (pg/ml)	0.003	0.003	1.265	0.261	1.003(0.998-1.008)
Basal P (ng/ml)	0.754	0.166	20.587	<0.01^*^	2.125(1.535-2.943)
AMH(ng/ml)	-0.263	0.029	83.813	<0.01^*^	0.769(0.727-0.813)
Total AFC	0.091	0.009	102.594	<0.01^*^	1.096(1.077-1.115)
Gn initial dose/1000 (IU)	0.496	0.937	0.28	0.596	1.642(0.262-10.298)
Gn total dose/1000 (IU)	0.069	0.063	1.174	0.279	1.071(0.946-1.213)
Gn duration (day)	0.053	0.03	3.128	0.077	1.054(0.994-1.117)
hCG day LH (IU/L)	-0.084	0.023	13.427	<0.01^*^	0.92(0.88-0.962)
hCG day E2 /1000(pg/ml)	0.356	0.027	179.522	<0.01^*^	1.427(1.355-1.503)
Endometrial thickness (mm)	-0.03	0.021	1.982	0.159	0.97(0.93-1.012)
No. Of retrieved oocytes	0.188	0.014	183.108	<0.01^*^	1.207(1.175-1.24)

*P < 0.05 is considered to be statistically significant.

OR, odds ratio.

### 3.3 Multivariate logistic regression

All variables associated with increased serum P levels on HCG days (derived from univariate analysis) were included. Multivariate logistic regression analysis was further performed to determine the predictors of increased serum P levels on HCG days. Finally, it was concluded that BMI, bP, serum E2, and LH levels on HCG day and the total number of oocytes retrieved were significantly related to the increase in serum P level on HCG day, as shown in **Table 3**.

**Table 3:** Multivariate logistic regression analysis of predictors of elevated serum progesterone levels on HCG day.

	P	OR(95%CI)
Maternal age (y)	0.883	1.002(0.973-1.032)
BMI (kg/m2)	<0.01^*^	0.901(0.862-0.941)
Type of infertility	0.183^*^	1.235(0.905-1.685)
Basal FSH (IU//L)	0.338	0.976(0.928-1.026)
Basal P (ng/ml)	<0.01^*^	2.1(1.439-3.066)
AMH(ng/ml)	0.703	1.016(0.936-1.103)
Total AFC	0.772	0.996(0.97-1.023)
hCG day E2 /1000(pg/ml)	<0.01^*^	1.225(1.146-1.31)
hCG day LH (IU/L)	0.032^*^	1.048(1.004-1.093)
No. Of retrieved oocytes	<0.01^*^	1.143(1.095-1.194)

*P < 0.05 is considered to be statistically significant.

OR, odds ratio.

There were no significant differences in implantation rate (45.91% *vs*. 41.49%, P = 0.433) or clinical pregnancy rate (40.95% *vs*. 35.11%, P = 0.292) between the NP and HP groups. However, the miscarriage rate was significantly higher in the NP group than in the HP group (7.97% *vs*. 2.13%, P = 0.043) (**Table 4**).

**Table 4:** Comparison of pregnancy outcomes between the two groups.

	NP group	HP group	*P*
Implantation rate(%)	45.91	41.49	0.433
Clinical pregnancy rate(%)	40.95	35.11	0.292
Miscarriage rate(%)	7.97	2.13	0.043*

*P < 0.05 is considered to be statistically significant.

## 4 Discussion

While freeze-all strategies with frozen embryo transfer (FET) have become increasingly prevalent in many centers—driven by advantages in endometrial-embryo synchronization and reduced ovarian hyperstimulation syndrome risk—fresh embryo transfer remains widely practiced globally, particularly in regions where cryopreservation infrastructure is limited or costs are prohibitive. Moreover, identifying predictors of hCG-day progesterone elevation retains clinical value even in freeze-all protocols: it informs personalized decisions about transfer strategy, guides stimulation adjustments to prevent premature progesterone rise, and may have implications for endometrial status in subsequent FET cycles. Thus, understanding these factors benefits patient care regardless of the ultimate transfer approach.

The human endometrium is affected by estrogen and progesterone secreted by the ovary and changes cyclically. It is divided into three stages: the menstrual phase, the secretory phase, and the proliferative phase. In 1934, Professors Adolf F. J. Butenandt and Schmidt converted pregnadiol into progesterone for the first time, laying the foundation for our subsequent research in the field of female reproduction (13). Progesterone is a steroid hormone that mainly acts on the endometrium through PR-A and PR-B receptors, converting the proliferative phase of the endometrium into the secretory phase of the endometrium (14). The endometrium in the secretory phase has more blood vessels, glands, and rich nutrients, which promote the maturity of the endometrium and allow the zygote to implant.

### 4.1 Clinical considerations for progesterone assessment

The timing and methodology of progesterone measurement merit careful consideration in clinical practice. In our study, serum progesterone was assessed on the day of hCG administration—a standard time point that captures the late-follicular phase rise preceding ovulation. However, alternative assessment strategies have been proposed. Some investigators advocate for measurement on the day of progesterone supplementation initiation in FET cycles, as this time point may better reflect endometrial preparation adequacy. Others have suggested serial monitoring in the late follicular phase to capture dynamic changes rather than a single threshold value.

Several technical factors influence progesterone measurement accuracy. Immunoassay-based methods, including the chemiluminescence assay used in our study (Beckman DIX-800), are susceptible to cross-reactivity with synthetic progestins and may exhibit variability between platforms. Liquid chromatography-tandem mass spectrometry (LC-MS/MS) offers superior specificity but is less practical for routine clinical use. Clinicians should be aware of their laboratory’s assay characteristics and establish center-specific thresholds, as the optimal “cut-off” value may vary by methodology and population.

Notably, the threshold of 1.5 ng/ml used in our study aligns with previous large-scale analyses (Venetis et al., 2013; Bosch et al., 2010), but some investigators have advocated for lower thresholds (0.8-1.0 ng/ml) or patient-specific thresholds based on individual ovarian response. Future prospective studies comparing different assessment strategies—including timing, frequency, and assay methods—would help establish evidence-based guidelines for progesterone monitoring in ART cycles.

It is well known that endometrial receptivity and embryo quality are key to the success of assisted reproductive technology (15). Endometrial receptivity refers to the ability of the endometrium to accept embryos, also known as the “window of implantation” (WOI) of the endometrium. It is regulated by a variety of factors and has strict time and space restrictions. The length of time is 30–36 hours, generally occurring between the 6th day (LH + 6) and the 9th day (LH + 9) after the LH surge in the natural cycle, or progesterone administration in the hormone replacement therapy (HRT) cycle between the 4th day (P + 4) and the 7th day (P + 7) (16).

Studies have shown (17–19) that premature elevation of progesterone levels in the late follicular phase will reduce the pregnancy rate of IVF-ET/ICSI, which is mainly thought to be related to asynchronous endometrial-embryo development and reduced embryo quality. Most studies claim (20, 21) that the increase in serum P levels on hCG day will not lead to a decrease in embryo quality, but Li J et al. came to the opposite conclusion (22). 58 infertile patients were included in the study and divided into the P ≤ 0.5 ng/ml group, the 0.5 ng/ml < P < 1.5 ng/ml group, and the P ≥ 1.5 ng/ml group. It can be concluded that P levels ≥ 1.5 ng/ml on the hCG day did not negatively impact embryonic morphokinetic parameters, instead resulting in faster embryo development in the initial stages (23).

The formation of high-quality embryos will significantly affect the pregnancy outcome of the next cycle using the embryo freezing strategy. Therefore, exploring the influencing factors of hCG daily serum P levels and early intervention is an important step to improve the pregnancy rate (24).

The incidence of high serum P level on hCG day in the antagonist regimen was higher than that in the agonist regimen (38% *vs* 35%) (9). Therefore, this study mainly includes people who undergo ovarian stimulation with antagonist regimens, and further explores the factors that affect the increase in serum P levels on hCG days in this population. The “cut-off value” of serum P levels on the hCG day of the current antagonist regimen is not clearly defined. According to previous relevant reports, “1.5ng/ml” was set as the “cut-off value” for dividing the two groups (25), and it was concluded that the bP level was positively correlated with the hCG day serum P level. A previous single-center retrospective cohort study included 1,472 patients (1,702 cycles) who underwent ovarian stimulation with an antagonist regimen. They were also divided into two groups using “1.5ng/ml” as the cut-off value, indicating that female age, bFSH, and bP levels can significantly predict serum P levels on hCG day (26). When adjusting for variables related to ovarian stimulation intensity (total number of days of Gn, total Gn dose, number of follicles >11mm on hCG day, serum estrogen level on hCG day), bP level is found to be the most predictive. hCG day serum P level was the only variable (OR: 6.30 (3.35–11.82)). This study also further analyzed patients who underwent multiple ovarian stimulation cycles (a total of 197 cases). Patients with a history of elevated serum P levels on hCG day were closely associated with increased serum P levels on hCG day of the next cycle (OR: 6.26 (1.81–21.62), and the risk of increased serum P levels on hCG day of the next cycle will be significantly increased (from 4.3% to 27.3%) (27). Similarly, Papaleo and Mutlu also concluded that bP can be used to predict progesterone levels on hCG day (28, 29). In addition, there are related reports that the ratio of hCG daily serum P level to mature oocyte number (POI) is also an effective indicator that can predict clinical pregnancy rate/live birth rate (30), and the threshold for obtaining the best pregnancy outcome is 0.36 (ng/ml/piece) (31).

This study found that BMI was negatively correlated with hCG day serum P levels. This may be because the ovaries of patients with low BMI are more sensitive to Gn, and the FSH and LH receptor activity in granulosa cells is higher, so more P is synthesized (32). It is also possible that obese women have significantly higher levels of oxidized low-density lipoprotein, which can act on corresponding receptors to increase the level of reactive oxygen species in the body, leading to oxidative stress (33, 34). Ultimately, it leads to increased apoptosis of granulosa cells in follicular cells (35, 36), which reduces progesterone secretion. However, the study sample size is small, and some confounding bias cannot be controlled; further confirmation with a larger number of samples is still needed. However, Tsai et al. came to the opposite opinion. There was a positive correlation between body weight and hCG day serum P level, with an OR value ranging from 0.94 to 0.96. However, the impact of BMI was not calculated and still needs further research (37). This may be because the higher the BMI, the greater the dose of Gn used during ovarian stimulation, the longer the stimulation time, and the sensitivity of granulosa cells to LH increases (38). Eventually, the conversion of cholesterol into progesterone increases, and a large number of follicles grow and develop, producing a large amount of E2 (39). At the same time, the granulosa cells produce progesterone, which enters the ovarian veins, ultimately leading to an increase in serum P levels (12). This study also found that the serum E2 level on hCG day was a risk factor for the serum P level on hCG day. Li J et al. also found that the serum E2 level on hCG day was positively correlated with the serum P level on hCG day. High doses of E2 interfere with the development of cleavage stage embryos, damage the endometrium-embryo interface, and interfere with embryo implantation. Therefore, clinically, we can try to adjust the dosage of exogenous FSH and LH in the middle and late stages of follicles according to the patient’s specific conditions, control the E2 and P levels on HCG day, and try to avoid the negative effects caused by excessive P levels on HCG day (40, 41). We also believe that the hCG day serum P level is positively correlated with the number of oocytes retrieved (OR: 1.141 (1.093-1.191)) (42), which is consistent with previous research (43). Progesterone is the end product secreted by granulosa cells, mainly metabolized by the liver, and has an accumulation effect in mature oocytes. Therefore, the greater the number of oocytes obtained, which exceeds the liver load, the higher the progesterone level in the late follicular phase (44).

Haouzi et al. (16) studied 15 COH patients, measured serum P levels according to the patient’s hCG day, and divided them into a P < 1.5ng/ml group and a P > 1.5ng/ml group. The endometrium was biopsied on day hCG+2 (the day of egg retrieval) and hCG+5 (during embryo transfer), and the endometrial gene expression profile was analyzed. It was concluded that there were temporal differences in gene expression of some genes between the two groups. The P>1.5ng/ml group may exhibit premature endometrial maturation and advance the “implantation window”, thereby interfering with embryo implantation and affecting the embryo implantation rate at the cleavage stage (45). It is worth mentioning that high levels of hCG day P also interfere with embryo implantation at the blastocyst stage and reduce the IVF-ET live birth rate.

HCG is a glycoprotein with a relative molecular weight of 36,700 and consists of two subunits, α and β. As follicles develop and mature, the concentration of FSH and LH receptors on granulosa cells and theca cells increases. The α subunit structure of HCG and LH (Luteinizing Hormone) is the same, and the β subunit structure is similar, that is, hCG and LH have the same hCG/LH receptor, which is called HCG/LH receptor complex (HCG/LH-R, also called LHCGR), therefore the use of hCG may induce increased production of P (46). The type and dosage of hCG were not included in this study. Therefore, further research on the clinical relationship between hCG use and hCG daily serum P levels is still needed in the future. Some studies also believe that the increase in serum P level on hCG day may be related to the response to ovulation induction drugs (Gn duration, Gn total dose, serum E2 level on hCG day) (22, 37), but this was not found in this study. Therefore, large-sample, multi-center, and prospective studies are still needed to further confirm the influencing factors of the increase in serum P levels on hCG days during ovulation induction with gonadotropin-releasing hormone antagonists.

In clinical practice, it is very important to control the BMI of infertile patients before ovarian stimulation (47). It can not only reduce the use of Gn dose, prevent the early appearance of LH peak, reduce the occurrence of OHSS, but also prevent premature increase in hCG daily P level (48). Therefore, appropriate weight loss is a good choice for obese patients. For infertile patients whose hCG day P levels are too high, most clinicians choose to cancel the transplant or perform whole-embryo freezing and elective frozen-thaw embryo transfer (49, 50). It has also been reported that dexamethasone reduces serum P levels by inhibiting the secretion of adrenocorticotropic hormone. Contraceptive pills can also inhibit endogenous gonadotropin secretion, functional ovarian cyst formation, and reduce basal P levels. Therefore, to avoid excessively high basal P levels, clinicians can appropriately use contraceptive pills for pretreatment to adjust patients’ serum P levels so that patients can benefit to the greatest extent (51).

In the present study, the total number of oocytes retrieved was identified as an independent risk factor for progesterone elevation on the day of hCG administration (OR = 1.143, p< 0.05). This finding aligns with the concept that progesterone is primarily secreted by granulosa cells as a terminal product of steroidogenesis. As the number of mature follicles increases, the cumulative granulosa cell mass expands, leading to higher total progesterone output, particularly when hepatic clearance capacity is exceeded. Moreover, patients with high ovarian reserve or hyper-responsiveness often exhibit enhanced luteinizing hormone (LH) receptor expression and increased steroidogenic activity, further contributing to progesterone accumulation. Notably, this relationship is clinically relevant because high ovarian response is often accompanied by elevated serum estradiol levels, which synergistically affect endometrial receptivity. Therefore, the number of retrieved oocytes serves not only as a marker of ovarian responsiveness but also as a direct indicator of the total steroidogenic burden. Clinicians should consider this factor when assessing the risk of premature progesterone rise, especially in patients with polycystic ovary morphology or high antral follicle counts, and may consider strategies such as cycle segmentation or elective frozen-thawed embryo transfer to circumvent the potential negative impact on endometrial–embryo synchronization.

### 4.2 Relevance of findings to frozen embryo transfer cycles

While our study was conducted in fresh embryo transfer cycles using a GnRH-antagonist protocol, the findings have important implications for frozen embryo transfer (FET) cycles as well. It is well recognized that the etiology of elevated progesterone levels differs between these two settings. In fresh cycles, late-follicular phase progesterone elevation arises endogenously from granulosa cells of developing follicles in response to gonadotropin stimulation. In FET cycles, elevated progesterone levels typically result from exogenous progesterone administration during endometrial preparation. Despite these distinct mechanisms, the consequences of progesterone elevation on endometrial biology are remarkably similar across both protocols.

Elevated serum progesterone—whether endogenous or exogenous—acts on endometrial progesterone receptors (PR-A and PR-B), accelerating the transition from proliferative to secretory endometrium. When progesterone levels rise prematurely or exceed physiological thresholds, the “implantation window” (WOI) is displaced and may close prematurely. This phenomenon has been well documented in both fresh cycles and FET cycles. Consequently, implantation failure rates increase regardless of whether the embryo is transferred fresh or frozen.

Importantly, the predictors of progesterone elevation identified in our study—AMH, basal progesterone, hCG-day serum E2 and LH levels, total oocyte yield, and BMI—are specific to the fresh cycle context, where progesterone production is driven by ovarian response. In FET cycles, elevated progesterone is primarily a function of exogenous dosing, route of administration (intramuscular, vaginal, subcutaneous), and individual patient metabolism. However, the protective role of higher BMI observed in our study may have parallels in FET cycles, as adipose tissue can affect steroid hormone distribution and clearance.

From a clinical perspective, our results can inform decision-making even when a freeze-all strategy is planned. For patients identified as high-risk for progesterone elevation (e.g., those with elevated basal progesterone, high E2 response, or high oocyte yield), clinicians may choose to avoid fresh transfer and proceed with elective FET. Moreover, understanding the factors that drive endogenous progesterone rise can help tailor stimulation protocols—such as adjusting Gn dosing or triggering method—to mitigate progesterone elevation, thereby potentially improving endometrial receptivity in the subsequent FET cycle. Thus, while the immediate clinical application differs, the underlying physiological principles remain relevant across fresh and frozen transfer paradigms.

## 5 Conclusion

Basal progesterone, serum E2 and LH levels on HCG day, and the total number of oocytes retrieved are independent risk factors for elevated serum progesterone on HCG day, while BMI is a protective factor. Understanding the factors that drive endogenous progesterone rise can help tailor stimulation protocols.

## Data Availability

The raw data supporting the conclusions of this article will be made available by the authors, without undue reservation.
